# Changes in Growth and Chemical Composition of the Essential Oil from Flowers and Leafy Stems of *Lavandula angustifolia* Grown in Media Amended with Bark and Sewage Sludge

**DOI:** 10.3390/molecules30234545

**Published:** 2025-11-25

**Authors:** Agnieszka Zawadzińska, Aneta Wesołowska, Ewa Skutnik, Julita Rabiza-Świder, Piotr Salachna

**Affiliations:** 1 Department of Horticulture, West Pomeranian University of Technology in Szczecin, Słowackiego 17 Str., 71-434 Szczecin, Poland; piotr.salachna@zut.edu.pl; 2 Department of Organic and Physical Chemistry, Faculty of Chemical Technology and Engineering, West Pomeranian University of Technology in Szczecin, Piastów Ave. 42, 71-065 Szczecin, Poland; aneta.wesolowska@zut.edu.pl; 3 Section of Ornamental Plants, Institute of Horticultural Sciences, Warsaw University of Life Sciences, Nowoursynowska 166, 02-787 Warsaw, Poland; ewa_skutnik@sggw.edu.pl (E.S.); julita_rabiza_swider@sggw.edu.pl (J.R.-Ś.)

**Keywords:** lavender, aromatic plant, essential oil, organic substrate, GC-MS

## Abstract

The growing medium is one of the key factors determining the yield and quality of lavender oil. The research conducted in greenhouse conditions aimed to assess the impact of a substrate with a reduced peat content enriched with compost from sewage sludge and bark on the growth, yield, and chemical composition of the oil from the inflorescences and leafy stems of English lavender ‘Sentivia Blue’. The plants were grown in pots filled with peat and chemical fertilizer, or in a substrate containing bark and sewage sludge compost, with or without fertilizer. Media affected the growth, leaf greenness index, and biomass production of lavender. Plants growing in peat with fertilizer were the tallest and widest. In turn, the highest number of inflorescences and the highest dry weight of inflorescences and leafy stems were found in plants grown in a mixture of bark and sewage sludge compost, with the addition of fertilizer. A significant interaction between the plant organ and the type of substrate was demonstrated, which affected the content of specific oil components. The content of essential oil was higher in inflorescences (1.15%) than in leaves (0.21%). The oil from the inflorescences was dominated by linalool, caryophyllene oxide, and linalyl acetate, while caryophyllene oxide, borneol, and geranyl acetate dominated in the leafy stems. The highest linalool content was found in oil obtained from inflorescences of plants grown in both media, based on bark and sewage sludge compost. The results show that the best quality parameters of the raw material and oil, including particularly high dry weight and linalool content, were obtained when the plants were grown in a medium consisting of bark, sewage sludge compost, and chemical fertilizer.

## 1. Introduction

The genus *Lavandula* L. (Lamiaceae) comprises approximately 39 species of herbaceous plants, subshrubs, and shrubs known for their oil-producing, medicinal, and ornamental properties [1]. The best-known species is English lavender *(Lavandula angustifolia* Mill., syn. *Lavandula officinalis* Chaix, *Lavandula vera* DC.), an evergreen shrub that occurs naturally in the mountainous regions of the Mediterranean Basin [2]. Lavender can synthesize essential oils, which are produced and accumulated as the end product of metabolism in glandular secretory hairs found mainly on flowers, leaves, and stems [3]. Lavender flower essential oil can contain about 300 chemical components, the most important of which are compounds from the monoterpene and sesquiterpene groups. The main compounds of the oil are: linalool, linalyl acetate, terpinen-4-ol, ocimene, acetate lavandulol, and cineole [4]. The profile of the oil from the flowers is best known, and there is little information available on the content and composition of the oil from the leaves and stems of lavender [5]. The descriptive and analytical composition of lavender oil varies depending on the variety or chemical race of the plant, the part of the plant, geographical origin, climatic conditions, stage of vegetation, place of cultivation, substrate, propagation, and time of harvest of the raw material [6,7,8,9,10,11].

It is estimated that global production of English lavender (*L. angustifolia)* essential oil is 300–500 tons per year [12]. The lavender oil market is projected to reach approximately $339.3 million in 2025 and is expected to grow to approximately $483.3 million by 2035. The leading producers of lavender oil include Bulgaria, France, Spain, Great Britain, China, Ukraine, Morocco, and India [13]. The cultivation of English lavender for processing is also becoming increasingly popular among Polish farmers. The total area of lavender cultivation in Poland is approximately 132.6 ha in 2024. Compared to 2022, when lavender was grown on an area of ~103.7 ha, this increase is as much as 28% [14]. The lavender market is showing an upward trend, driven by growing consumer interest in a product with a pure composition, a wide range of applications, and a natural, certified origin [14,15]. Therefore, it is important to seek environmentally friendly methods of lavender cultivation using sustainable agricultural practices that allow for the production of high-quality raw materials [16,17].

Municipal sewage sludge is a waste product generated on a large scale worldwide, constituting a rich source of organic matter and nutrients. In Poland, the amount of sewage sludge produced in municipal sewage treatment plants in 2023 amounted to 9465 thousand tons of dry solid [18]. In light of European Union regulations, Poland has committed to utilizing all of it in the coming years, including for agricultural purposes [19]. Composting sewage sludge with various types of structure-forming materials, during which the organic matter is sanitized and stabilized, is one of the cheapest and most rational ways of treating it. By using compost in appropriate proportions with peat and other additives, e.g., composted bark, substrate mixtures with properties adapted to the requirements of specific plant species can be obtained [20,21]. The use of sewage sludge compost may be a promising approach in the cultivation of oil-bearing plants [22,23,24]. The limited data available indicate that sewage waste can be used in specific doses as a valuable source of organic matter, stimulating oil production in the cultivation of English lavender [25,26]. However, the effect of media amended with sewage sludge compost on the quality of English lavender oil is not yet known.

The standard medium used in soilless cultivation is peat enriched with chemical compound fertilizers. Peat extraction is associated with enormous losses to the environment. Pro-ecological strategies assume the reduction and even complete withdrawal of peat from plant production in the future. There is therefore a need to develop new media to replace peat. The objective of the present study was to evaluate the growth and chemical composition of the inflorescences and leafy stems essential oils from English lavender grown in media containing (by volume) 50% bark and 10% compost with sewage sludge, with and without additional chemical fertilization. Lavender ‘Sentivia Blue’, a cultivar with a previously unknown oil profile, was selected for the study. We hypothesized that peat substrate amended with bark and compost with sewage sludge is a promising substrate mix alternative for peat in lavender plant production.

## 2. Results

### 2.1. Plant Growth and Biomass Production

**Figure 1:**
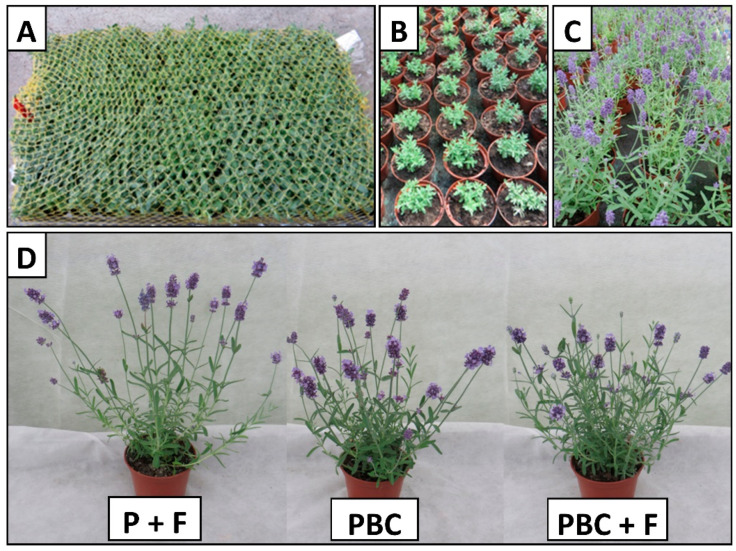
Appearance of English lavender ‘Sentivia Blue’ plants (**A**) right after delivery from the nursery; (**B**) on the 14th day after planting (**C**) on the 62nd day after planting (**D**) comparison of plants growing in media (P + F, peat + chemical fertilizer; PBC, 40% peat + 50% bark + 10% sewage sludge compost; PBC + F, 40% peat + 50% bark + 10% sewage sludge compost + chemical fertilizer).

Table 1 reports the effect of the growing media on English lavender ‘Sentivia Blue’ growth, leaf greenness, and biomass. Plant height and plant width were higher when plants were grown in pure peat with chemical fertilizer (P + F). In contrast, plants grown in a medium based on bark and sewage sludge compost with the addition of chemical fertilizer (PBC + F) had greener leaves and produced the most inflorescences compared to plants grown in peat (P + F), by 22.9% and 72.9%, respectively (Table 1, Figure 1).

Media affected the fresh weight of the inflorescence and the dry weights of the inflorescence and leafy stems (Table 1). The weights of both fresh and dried inflorescences were significantly reduced in plants grown in bark and compost-based substrate without fertilization (PBC) compared to the other two media. When plants were grown in a medium amended with bark, compost, and fertilizer (PBC + F), the dry weights of both inflorescences and leafy stems were the greatest among the treatments used. Compared to plants grown in peat (P + F), the dry weights of inflorescences and leafy stems of plants growing in PBC + F medium were increased by 11.8% and 6.4%, respectively (Table 1).

### 2.2. Essential Oil Content and Composition

Overall, the oil content in English lavender ‘Sentivia Blue’ inflorescences ranged from 1.01 ± 0.09% (PBC medium) to 1.23 ± 0.15% (PBC + F medium), while in leafy stems it ranged from 0.20 ± 0.02% (P + F and PBC + F media) to 0.24 ± 0.02% (PBC medium) (Table 2). Regardless of the medium used, the average oil content in inflorescences was 1.15 ± 0.12% and was 5.5 times higher than the average oil content in leafy stems (0.21 ± 0.02%).

Based on chromatographic analysis, a total of 122 compounds covering more than 99.4% were identified in lavender inflorescences, and a total of 133 compounds covering more than 99.3% of the total were identified in leafy stems (Supplement Tables S1 and S2).

Table 2 and Table 3 show the chemical profile of the oil of English lavender ‘Sentivia Blue’, taking into account all oil components with a content of more than 1% in at least.

Statistic results given in Table 3 show that essential oil composition varied by plant part, growing media and interaction between the plant organ and the type of medium. Table S3 reports the chemical analysis of English lavender ‘Sentivia Blue’ oil depending on plant part, regardless of the type of growing medium. The main components of the oil obtained from inflorescences were: linalool (16.63 ± 1.24%), caryophyllene oxide (11.91 ± 0.89%), linalyl acetate (8.80 ± 0.82%), cis-linalool oxide (5.06 ± 0.49%), and α-terpinolene (3.88 ± 0.45%) (Table S3). The content of these components in the oil from the inflorescences was significantly higher compared to their content in the oil obtained from leafy stems. The main components of the oil obtained from leafy stems were: caryophyllene oxide (10.94 ± 0.80%), borneol (7.81 ± 1.36%), geranyl acetate (5.74 ± 0.27%), τ-cadinol (5.38 ± 0.74%), and linalool (3.85 ± 0.37%). Among the listed components, the content of borneol, geranyl acetate, and τ-cadinol was significantly higher compared to their content in the oil obtained from inflorescences (Table S3).

Table S4 shows the chemical analysis of English lavender ‘Sentivia Blue’ oil depending on the type of medium, regardless of the plant organs evaluated. Significant changes were found in the content of cis-linalool oxide, α-terpinolene, linalool, 1,2-dihydrolinalool, borneol, α-terpineol, lavandulyl acetate, α-santalene, γ-cadinene, β-sesquiphellandrene, caryophyllene oxide, α-eudesmol, and cadalene depending on the type of medium. In the case of the remaining oil components, no effect of the media on their composition was observed. The oil obtained from plants grown in peat, along with the addition of chemical fertilizer (P + F), contained the highest amounts of cis-linalool oxide (3.00 ± 2.91%), α-terpinolene (2.24 ± 2.43%), borneol (6.56 ± 3.29%), and γ-cadinene (1.26 ± 0.65%). The oil obtained from plants grown in a medium amended with bark and compost without additional chemical fertilization (PBC) contained the highest amounts of 1,2-dihydrolinalool (2.30 ± 0.36%), α-terpineol (1.08 ± 0.49%), lavandulyl acetate (2.21 ± 1.40%), α-santalene (1.84 ± 0.80%), caryophyllene oxide (12.24 ± 0.72%), α-eudesmol (1.85 ± 0.63%), and cadalene (2.73 ± 0.16%). When the plants were grown in a medium contained bark and compost with the addition of chemical fertilizer (PBC + F substrate), the oil obtained from them contained the highest amounts of linalool (10.87 ± 7.78%) and β-sesquiphellandrene (1.26 ± 0.62%) (Table S4).

A significant interaction between the plant organ and the type of medium was demonstrated for the percentage content of cis-linalool oxide, α-terpinolene, linalool, α-terpineol, linalyl acetate, α-santalene, and β-sesquiphellandrene (Table 2 and Table 3). The highest content of cis-linalool oxide, α-terpinolene, and linalyl acetate was found in oil obtained from inflorescences of plants grown in peat with the addition of chemical fertilizer (P + F). In turn, the highest content of linalool and α-terpineol was found in the oil obtained from inflorescences of plants grown in both media amended with bark and compost (PBC and PBC + F). The highest content of α-santalene was found in oil from leafy stems obtained from plants grown in a medium containing bark and compost without fertilization (PBC). The highest β-sesquiphellandrene content was found in oil from leafy stems of plants grown in media with the addition of bark, compost, and chemical fertilizer (PBC and PBC + F) (Table 2).

## 3. Discussion

In lavender production, plant growth and yield depend mainly on the type of medium and its nutrient content [27,28]. For lavender and other oil-producing plants, media with reduced peat content are sought. At the same time, the use of chemical fertilizers in plant production is being reduced in favor of organic fertilizers [28,29]. In this study, English lavender ‘Sentivia Blue’ was grown in a medium with reduced peat content, based on compost containing bark and sewage sludge, with (PBC + F) or without the addition of chemical fertilizer (PBC). The reference object was pure peat amended with fertilizer (P + F). Both the sewage sludge compost itself and the two media containing it used in the study met the criteria for heavy metal and microbiological contamination content [30,31]. In our study, we used composted pine bark from sustainably managed forests as a substitute for peat. The addition of composted bark to the substrate has a beneficial effect on its structure, aeration, and water retention capacity [32]. The compost and bark-based media used in this study met the recommendations of lavender grower [33] regarding substrate pH and salinity.

English lavender plants developed typically throughout the entire cultivation period, did not show any drastic growth inhibition, and did not exhibit any symptoms of phytotoxicity in the form of visible chlorosis or necrosis (Figure 1). In peat substrate containing the recommended dose of chemical fertilizer (P + F), the plants were the tallest and widest. The increased vegetative growth of the plants may have resulted from the supply of nitrogen in the form of fertilizer that was easily and quickly absorbed by the roots in a pure peat environment with exceptional air and water properties [34]. This hypothesis is confirmed by the fact that plants grown in peat with fertilizer had a reduced dry weight of their above-ground parts. A large amount of nitrogen uptake leads to intensive growth of the vegetative parts of plants and increased water content in tissues [35,36], which in turn results in a reduction in dry weight.

Plants grown in a medium with added bark, compost, and fertilizer (PBC + F) had the highest leaf index, produced the most inflorescences, and had the highest dry weight of biomass compared to the other treatments (Table 1). It is worth noting that the dry weight of the raw material determines the yield of essential oils from plants. Similarly, other researchers report the beneficial effect of adding sewage sludge to the medium on the biomass of lavender and other oil-producing plant species. Seyedalikhani et al. [26] found that the application of sewage waste to degraded soil increased the dry biomass of *L. angustifolia* and *Rosmarinus officinalis*. Mazeed et al. [37] reported that adding sewage sludge to the soil increased the dry weight of stems in potted geraniums. The beneficial effect of the PBC + F medium on morphological characteristics demonstrated in these studies may have resulted from the high content of assimilable forms of phosphorus and potassium in the compost (Table S5), key macroelements that modify the growth and flowering of lavender [38,39]. In addition, the stimulating effect of the PBC + F medium on herb yield may have resulted from combining organic and inorganic fertilization, which improved nutrient availability and, consequently, plant productivity. Silva et al. [40] showed that organic-mineral fertilization in *Lavandula dentata* cultivation provides a higher lavender yield due to the slow release of nutrients than the use of mineral fertilizer alone.

The main goal of lavender cultivation is to obtain a high yield of essential oil, both in terms of quantity and quality. In this study, the content and profile of essential oils in the inflorescences and leafy stems of English lavender ‘Sentivia Blue’ were evaluated for the first time (Table 2). According to the breeder [33], the ‘Sentivia Blue’ cultivar is characterized by flowering in the first season of cultivation without the need for vernalization of plants and intensely fragrant flowers. There is no data on the suitability of this variety for cultivation for oil production. It turned out that the oil content in dried inflorescences of English lavender ‘Sentivia Blue’ was 1.15% and did not exceed the Polish standard requiring at least 1.7% oil [41]. According to Aprotosoaie et al. [1], who analyzed many research results, the average oil content in dried inflorescences/flowering aerial parts of *L. angustifolia* ranged from 0.5% to 9.62%, depending on the region of origin. Studies conducted in Poland show that the oil content of *L. angustifolia* inflorescences grown in the field was 1–2%, depending on the distillation time used [42]. In the case of leafy shoots, the average oil content of English lavender ‘Sentivia Blue’ was 0.21%. The same oil content (0.21%; *v*/*w*) in the dried leaves was found in *L. angustifolia* grown in Algeria [43]. The data presented show that the inflorescences of English lavender ‘Sentivia Blue’ contained significantly more oil than its leafy stems, which is consistent with other studies [5], where it was shown that lavender inflorescences are the main organ of essential oil accumulation. Wilson et al. [44] analyzed the yield and composition of oil in *L. angustifolia* across different parts of the plant, showing that the highest oil content was found in the corolla (0.1%) and the lowest in the leaf (0.05%). Similarly, Nurzyńska-Wierdak and Zawiślak [45] found that the oil content in *L. angustifolia* was highest in the inflorescences (3.2 mL 100 g^−1^), followed by the flower buds (2.7 mL 100 g^−1^) and leaves (0.6 mL 100 g^−1^).

The main component of the oil from English lavender ‘Sentivia Blue’ inflorescences was linalool, followed by caryophyllene oxide and linalyl acetate. In the case of oil from leafy stems, caryophyllene oxide was dominant (Table S3). Linalool and linalyl acetate have been identified as the main components of *L. angustifolia* oil obtained from flowers in many studies [45,46,47,48]. Linalool and linalyl acetate, which belong to the oxygenated monoterpene group, are volatile substances characterized by a floral, citrus, and sweet scent. For this reason, both compounds are used in the cosmetics and perfume industry, the food industry as flavorings, and household chemicals [49].

In this study, the composition of the tested oil from English lavender ‘Sentivia Blue’ inflorescences was found to have a high content of caryophyllene oxide (11.1–12.7%) compared to data from the literature, where the content of this component in *L. angustifolia* ranges from 1.8 to 6.69% [1,9,50,51]. In the case of oil from the leafy stems of English lavender ‘Sentivia Blue’, the caryophyllene oxide content was 10.2–11.8% and was similar to the results obtained for *L. angustifolia* ‘Munstead’, where the content of this oxygenated sesquiterpene was 8.54–12.23% [52]. Caryophyllene oxide has anti-inflammatory, antifungal, and antibacterial properties [53]. The compound has a spicy, woody-peppery aroma and is used in perfumes, lotions and creams, aromatherapy products, and as a preservative in food and medicines [54]. The high content of caryophyllene oxide as a chemotaxonomic feature can be used in practice to assess the quality of lavender oil. Pokajewicz et al. [47] propose to consider caryophyllene oxide as a marker distinguishing true lavender (*L. angustifolia)* oil from lavandin (*L.* × *intermedia)* oil. The composition of essential oils obtained from *L. angustifolia* flowers is specified by the requirements of the Pharmacopoeia and ISO, while there are no such standards for oils from leaves and stems [5]. Essential oils are characterized by significant variability in composition, which is influenced by many factors, including chemotype, growing conditions, harvest phase, drying and storage methods, oil extraction methods, and analytical parameters used to identify their compounds [5,50,54,55].

The type of medium used modified the chemical profile of English lavender ‘Sentivia Blue’ essential oils. The oil from inflorescences growing in media with added bark and compost had an increased content of linalool and α-terpineol. In the case of oil derived from leafy stems, the use of both bark- and compost-based substrates increased the content of β-sesquiphellandrene (Table 2). Studies on the content of individual components of *L. angustifolia* essential oil depending on the composition of the medium and the use of compost are inconclusive. Mavandi et al. [56] demonstrated changes in the content of oxygenate monoterpenes, including 1,8-cineole, borneol, and camphor, in the oil from the leaves of *L. angustifolia* grown in soil enriched with vermicompost and cow manure. In the case of *Lavandula dentata,* the use of organo-mineral fertilizer led to changes in the content of 1,8-cineole, fenchone, and camphor in the oil from flowers and leaves [40]. On the other hand, no changes in the qualitative composition of *L. angustifolia* essential oil obtained from inflorescences of plants growing in different media with the addition of compost [9] or conifer wood biochar [57] were found. The changes in the content of certain components of lavender oil, resulting from the use of sewage sludge compost, may be due to the introduction of specific elements into the medium. These elements are utilized by plants in the biosynthesis and accumulation of specialized metabolites that are part of the oils [57,58,59,60].

## 4. Materials and Methods

### 4.1. Plant Material, Experimental Design and Growing Conditions

Young plants of English lavender ‘Sentivia Blue’ from in vitro cultures, with an average height of 6.0 ± 1.0 cm, were obtained from the commercial perennial nursery Vitroflora (Trzęsacz, Poland), which specializes in plant material propagation.

Three media were used in the experiment (Table 4), which were prepared from three components with different chemical properties (Table 5), determined according to the methods [61,62]. In brief, the pH of growing media was determined in water extract (1:2; *v*:*v*) by the TESTER CP-505 m (Elmetron, Zabrze, Poland); salinity was measured using the CCP-401 conductometer with an EC-60 sensor (Elmetron, Zabrze, Poland); the content of available nitrate nitrogen in the media were determined by the potentiometric method; the available phosphorus content was determined by a spectrophotometric method, available potassium by flame photometry, and available magnesium and calcium by flame atomic absorption spectrometry.

Media were formulated using high peat (Klasmann-Delimann, Geeste, Germany), compost made from aerobically stabilized sewage sludge with the addition of shredded branches, leaves and mowed grass, and composted pine bark from trees uncontaminated by chemicals. The physicochemical properties of the compost obtained are presented in Table S5. In brief, the bulk density and humidity of compost were assessed according to European standards [63]. A Kjeldahl digestion kit and unit (Vapodest, Gerhardt GmbH, Konigswinter, Germany) were used to determine total nitrogen content by titration. Organic carbon was determined by Dumas’ method with the Carbon Sulfur Determinator CS-530 apparatus (Eltra, GmbH, Neuss, Germany). After wet mineralization of samples in a 65% HNO_3_ and 75% HClO_4_ mixture, the content of total forms of elements was determined by plasma spectrometry (Inductively Coupled Plasma Optical Emission Spectrometry) with the Optima 2000 DV sequential spectrometer (Perkin-Elmer, Boston, MA, USA).

The dose of compost was determined based on the results of a pilot study conducted under greenhouse conditions, in which out of three tested doses (10, 20 and 30% by volume), the 10% most favorably influenced the media salinity, plant height, plant width, leafy stem number per plant, leaf greenness index, plant fresh weight of lavender plants (Table S6, Table S7). Two media (P + F and PBC + F) amended with a chemical fertilizer Azofoska (Grupa Inco, Warsaw, Poland) the following composition: 13.6% N (5.5% N-NO_3_; 8.1% N-NH_4_), 2.8% P, 15.8% K, 2.7% Mg, 9.2% S, 0.045% B, 0.17% Fe, 0.27% Mn, 0.18% Cu, 0.045% Zn, 0.04% Mo). A fertilizer dose of 1.5 g dm^−3^ of medium recommended for bedding plants was used [33].

Lavender young plants were planted individually in plastic pots with a diameter of 14 cm (capacity 1.2 dm^3^) in three prepared media (Table 4), 20 plants in each. The plants were grown in a completely randomized design for 64 days in the greenhouse of the Department of Horticulture, West Pomeranian University of Technology in Szczecin (53°25′ N, 14°32′ E) during the summer, under natural photoperiod conditions. The average monthly maximum/mean/minimum air temperatures were, respectively: June 26.9 °C/19.8 °C/14.2 °C, July 27.5 °C/20.2 °C/14.4 °C, and August 28.7 °C/20.8 °C/14.8 °C. The average monthly maximum/mean/minimum relative humidity was, respectively: June 92.4%/72.3%/48.6%, July 94.8%/76.4%/51.5%, and August 91.5%/71.6%/44.5%. The plants were watered 1–2 times a week. The frequency of watering depended on the medium water potential, which was maintained at −30 kPa based on tensiometer readings (MMM Tech Support, Berlin, Germany).

The plants were not pruned, fertilized, or treated with any plant protection products during cultivation.

### 4.2. Morphological Measurements

Morphological measurements were performed at full bloom on 10 balanced plants in each treatment. Plant height (measured from the growing medium surface to the top of the plant), plant width (measured twice per plant at right angles; the two measurements were then averaged), the number of inflorescences per plant, and leaf greenness were determined by nondestructively measuring leaf SPAD chlorophyll index using a Chlorophyll Meter SPAD-502 (Minolta, Osaka, Japan). Two SPAD readings were taken on 10 randomly selected leaves, 10 plants in each treatment.

Lavender was harvested on the last day of cultivation, when the plants were in full bloom. Plants were cut at their bases. Fresh weights of inflorescences and leafy stems were recorded separately for each plant on a laboratory scale (PS 200/2000/C/2, RADWAG, Radom, Poland). The plant material was dried in the dark at a temperature of 30–32 °C. After drying, the dry weights of inflorescences and leafy stems were determined.

### 4.3. Essential Oils Analysis

#### 4.3.1. Essential Oil Extraction

Dried inflorescences (15 g) and leafy stems (20 g) of each sample were separately subjected to hydrodistillation in a Clevenger-type apparatus for 2 h, using 400 mL of distilled water, according to the method recommended by the European Pharmacopoeia [64]. The obtained essential oils were dried over anhydrous sodium sulphate, filtered, and stored in dark sealed vials at 4 °C until GC-MS analysis was performed. Essential oil content (%) was calculated as the volume (mL) of essential oil per 100 g of dry inflorescences or stems/leaves.

#### 4.3.2. Gas Chromatography-Mass Spectrometry (GC-MS)

The qualitative GC-MS analysis of the lavender oils was carried out using a Hewlett-Packard 6890 gas chromatograph equipped with a fused silica capillary column HP-5MS (5% phenylmethylsiloxane, 30 m × 0.25 mm, 0.25 μm film thickness, Agilent Technologies, USA) and coupled with a 5973 mass selective detector from the same company. The essential oil samples (30 mg) were dissolved in dichloromethane (1.5 mL), and 1 µL of each solution was injected in a split mode at a ratio of 5:1. The flow rate of helium (carrier gas) through the column was kept at 1.2 mL min^−1^. The initial temperature of the column was 45 °C, then increased to 200 °C at a rate of 5 °C min^−1^ (kept constant for 10 min), and then increased to a final temperature of 250 °C at a rate of 5 °C min^−1^. The oven was held at this temperature for 20 min. The injector temperature was 250 °C, the transfer line temperature was 280 °C, and the ion source temperature was 230 °C. The solvent delay was 4 min. MS conditions were as follows: ionization voltage of 70 eV, acquisition mass range 50–550, scan rate—2.94 scans/s. The total running time for a sample was 71 min.

The relative percentage of the essential oil constituents was evaluated from the total peak area (TIC) using the apparatus software. Essential oil constituents were identified by comparison of their retention indices (relative to n-alkanes C_7_-C_30_ on HP-5MS column) with those reported in NIST Chemistry WebBook [65] and literature [66]. Further identification was made by comparison of their mass spectra with those stored in the Wiley NBS75K.L and NIST/EPA/NIH (2002 version) mass spectral libraries using different search engines (PBM, Nist02).

### 4.4. Statistical Analysis

The results obtained from biometric measurements of plants were statistically analyzed using Tibco Statistica 13.3 software (StatSoft, Krakow, Poland) with a one-way analysis of variance. The results of the chemical composition of oils were analyzed using a two-way analysis of variance (3 growing media × 2 plant parts). Differences between means were compared using Tukey’s honestly significant difference at *p* ≤ 0.05.

## 5. Conclusions

This study indicates that media amended with bark and compost based on sewage sludge proved useful in pot cultivation of lavender and ensured a high-quality herb yield. Application of medium containing bark and compost with additional chemical fertilizer (PBC + F) achieved improvements in the accumulation of dry weight in the above-ground parts and linalool concentration in the inflorescences oil in lavender. English lavender ‘Sentivia Blue’ inflorescences contained 5.5 times more oil than leafy stems. The major compound was linalool, caryophyllene oxide, and linalyl acetate in inflorescences and caryophyllene oxide in leafy stems. In summary, the appropriate choice of medium can modify the yield of the herb and the quality of the essential oil in lavender.

## Figures and Tables

**Table 1:** Effect of the growing media on plant characteristic and biomass of English lavender ‘Sentivia Blue’. The data are presented as mean ± SD. Peat + chemical fertilizer (P + F); 40% peat + 50% bark + 10% sewage sludge compost (PBC); 40% peat + 50% bark + 10% sewage sludge compost + chemical fertilizer (PBC + F).

Parameters	Growing Media		
	P + F	PBC	PBC + F
Plant height (cm)	35.2 ± 0.93 a	25.3 ± 0.87 b	27.3 ± 1.20 b
Plant width (cm)	36.0 ± 2.16 a	28.7 ± 2.79 c	31.1 ± 1.16 b
Inflorescences (no./plant)	13.3 ± 0.58 c	18.7 ± 1.53 b	23.0 ± 2.00 a
Leaf greenness (SPAD)	54.5 ± 1.32 b	56.1 ± 1.63 b	67.0 ± 1.29 a
Inflorescence fresh weight (g/plant)	8.11 ± 0.11 a	6.47 ± 0.49 b	8.07 ± 0.10 a
Leafy stems fresh weight (g/plant)	32.2 ± 0.41 a	34.2 ± 1.74 a	33.1 ± 0.27 a
Inflorescence dry weight (g/plant)	2.28 ± 0.07 b	1.88 ± 0.04 c	2.55 ± 0.08 a
Leafy stems dry weight (g/plant)	5.60 ± 0.04 b	5.71 ± 0.03 b	5.96 ± 0.04 a

Values in raw with different letters show statistically significant differences based on ANOVA and Tukey test at *p* ≤ 0.05.

**Table 2:** The interaction effects of plant part × growing media on the chemical composition (%) of the essential oil from English lavender ‘Sentivia Blue’. Peat + chemical fertilizer (P + F); 40% peat + 50% bark + 10% sewage sludge compost (PBC); 40% peat + 50% bark + 10% sewage sludge compost + chemical fertilizer (PBC + F).

Compound	Rt [min]	RI	Inflorescence			Leafy stem		
			Growing Media			Growing Media		
			P + F	PBC	PBC + F	P + F	PBC	PBC + F
Essential oil content % (*v*/*w*)			1.20 ± 0.12	1.01 ± 0.09	1.23 ± 0.15	0.20 ± 0.02	0.24 ± 0.02	0.20 ± 0.02
β-Pinene	7.27	974	1.40 ± 0.03	1.21 ± 0.08	1.16 ± 0.11	3.74 ± 0.07	3.09 ± 1.21	2.98 ± 0.45
m-Cymene	8.47	1020	0.41 ± 0.03	0.44 ± 0.03	0.39 ± 0.05	1.12 ± 0.06	1.12 ± 0.25	1.23 ± 0.13
p-Cymene	8.54	1022	0.79 ± 0.04	0.85 ± 0.05	0.74 ± 0.08	2.23 ± 0.15	2.34 ± 0.56	2.37 ± 0.31
Eucalyptol	8.71	1028	1.11 ± 0.04	1.23 ± 0.07	1.39 ± 0.09	1.53 ± 0.16	1.32 ± 0.35	1.40 ± 0.19
*cis*-Linalool oxide	9.85	1069	5.52 ± 0.04 a	4.49 ± 0.25 b	5.17 ± 0.13 a	0.48 ± 0.01 c	0.53 ± 0.06 c	0.63 ± 0.07 c
α-Terpinolene	10.30	1084	4.35 ± 0.21 a	3.39 ± 0.18 b	3.93 ± 0.18 ab	0.14 ± 0.01 c	0.12 ± 0.02 c	0.16 ± 0.03 c
Linalool	10.77	1101	15.17 ± 0.04 b	17.13 ± 0.99 a	17.60 ± 0.21 a	3.77 ± 0.13 c	3.64 ± 0.24 c	4.15 ± 0.57 c
1,2-Dihydrolinalool	11.73	1137	1.65 ± 0.19	2.00 ± 0.05	1.67 ± 0.18	2.34 ± 0.16	2.61 ± 0.15	2.23 ± 0.20
Camphor	11.87	1142	0.84 ± 0.02	0.81 ± 0.03	0.82 ± 0.04	1.89 ± 0.09	1.52 ± 0.15	1.57 ± 0.32
Pinocarvone	1239	1161	0.96 ± 0.05	1.00 ± 0.04	0.86 ± 0.05	2.35 ± 0.18	2.29 ± 0.25	2.12 ± 0.39
Borneol	12.55	1167	3.73 ± 0.01	3.26 ± 0.12	3.41 ± 0.05	9.38 ± 0.75	7.13 ± 0.49	6.92 ± 1.01
p-Cymen-8-ol	12.95	1182	0.71 ± 0.09	0.68 ± 0.01	0.67 ± 0.10	1.04 ± 0.03	1.33 ± 0.01	1.30 ± 0.28
α-Terpineol	13.18	1190	1.05 ± 0.02 b	1.51 ± 0.04 a	1.50 ± 0.02 a	0.60 ± 0.04 c	0.65 ± 0.04 c	0.62 ± 0.16 c
Myrtenol	13.34	1196	1.68 ± 0.04	1.82 ± 0.05	1.62 ± 0.04	2.93 ± 0.23	2.91 ± 0.23	2.59 ± 0.37
Verbenone	13.67	1208	1.46 ± 0.10	1.42 ± 0.01	1.37 ± 0.06	1.26 ± 0.06	1.55 ± 0.07	1.40 ± 0.28
Bornyl formate	14.20	1228	0.26 ± 0.06	0.36 ± 0.00	0.31 ± 0.08	1.13 ± 0.07	1.04 ± 0.04	1.06 ± 0.27
Linalyl acetate	14.94	1255	9.59 ± 0.56 a	8.01 ± 0.04 a	8.79 ± 0.75 a	1.47 ± 0.01 b	1.77 ± 0.08 b	1.93 ± 0.37 b
Lavandulyl acetate	15.89	1290	3.07 ± 0.03	3.39 ± 0.06	3.20 ± 0.13	0.77 ± 0.02	1.02 ± 0.07	1.09 ± 0.12
Geranyl acetate	18.35	1384	1.44 ± 0.08	1.77 ± 0.27	1.47 ± 0.09	5.93 ± 0.15	5.62 ± 0.28	5.69 ± 0.42
α-Santalene	19.28	1421	1.33 ± 0.05 c	1.16 ± 0.12 cd	0.94 ± 0.01 d	2.19 ± 0.04 b	2.53 ± 0.06 a	2.14 ± 0.08 b
γ-Cadinene	21.61	1515	0.69 ± 0.03	0.48 ± 0.05	0.37 ± 0.01	1.82 ± 0.08	1.61 ± 0.01	1.61 ± 0.04
β-Sesquiphellandrene	21.72	1520	0.74 ± 0.03 c	0.81 ± 0.12 c	0.73 ± 0.01 c	1.36 ± 0.06 b	1.62 ± 0.06 a	1.80 ± 0.05 a
Elemol	22.52	1554	1.11 ± 0.06	1.32 ± 0.13	1.17 ± 0.04	1.14 ± 0.08	1.30 ± 0.14	1.25 ± 0.06
Caryophyllene oxide	23.31	1588	11.1 ± 0.69	12.7 ± 0.74	11.9 ± 0.57	10.2 ± 0.38	11.8 ± 0.35	10.9 ± 0.66
τ-Cadinol	24.58	1644	2.11 ± 0.17	1.78 ± 0.07	1.54 ± 0.18	5.64 ± 0.49	4.91 ± 0.28	5.61 ± 1.33
α-Eudesmol	24.92	1659	2.13 ± 0.01	2.39 ± 0.03	2.10 ± 0.18	0.90 ± 0.10	1.31 ± 0.13	1.28 ± 0.16
Cadalene	25.23	1673	2.08 ± 0.18	2.80 ± 0.04	2.50 ± 0.16	2.25 ± 0.25	2.67 ± 0.25	2.21 ± 0.30
epi-α-Bisabolol	25.60	1689	0.61 ± 0.02	0.45 ± 0.01	0.57 ± 0.05	1.52 ± 0.16	1.40 ± 0.21	1.53 ± 0.88

The data are presented as mean ± SD. Values in raw with different letters show statistically significant differences based on ANOVA and Tukey test at *p* ≤ 0.05. Rt—retention time; RI—retention indices relative to n-alkanes (C7–C30) on HP-5 MS capillary column.

**Table 3:** ANOVA *p*-values showing the effect of plant part and growing media on the essential oils of English lavender ‘Sentivia Blue’.

Compound	Plant Part	Growing Media	Plant Part × Growing Media
β-Pinene	***	ns	ns
m-Cymene	***	ns	ns
p-Cymene	***	ns	ns
Eucalyptol	ns	ns	ns
*cis*-Linalool oxide	***	**	**
α-Terpinolene	***	**	**
Linalool	***	*	*
1,2-Dihydrolinalool	**	*	ns
Camphor	***	ns	ns
Pinocarvone	***	ns	ns
Borneol	***	*	ns
p-Cymen-8-ol	***	ns	ns
α-Terpineol	***	**	*
Myrtenol	***	ns	ns
Verbenone	ns	ns	ns
Bornyl formate	***	ns	ns
Linalyl acetate	***	ns	*
Lavandulyl acetate	***	**	ns
Geranyl acetate	***	ns	ns
α-Santalene	***	**	**
γ-Cadinene	***	***	ns
β-Sesquiphellandrene	***	**	**
Elemol	ns	ns	ns
Caryophyllene oxide	*	*	ns
τ-Cadinol	***	ns	ns
α-Eudesmol	***	*	ns
Cadalene	ns	*	ns
epi-α-Bisabolol	**	ns	ns

ns, ***, **, *, Nonsignificant or significant F test at *p* ≤ 0.001, 0.01 or 0.05, respectively.

**Table 4:** Growing media in with English lavender ‘Sentivia Blue’ were grown.

Growing Media	Name
100% peat + chemical fertilizer	P + F
40% peat + 50% bark + 10% sewage sludge compost	PBC
40% peat + 50% bark + 10% sewage sludge compost + chemical fertilizer	PBC + F

**Table 5:** Chemical characteristics of growing media.

Parameters	Peat (P)	Bark (B)	Compost (C)	PBC
pH (H_2_O, 1:2, *v*:*v*)	6.40	5.20	7.50	6.30
Salinity (g NaCl dm^−3^)	0.29	0.15	3.92	0.84
NO_3_-N (mg dm^−3^)	7	5	290	16
P (mg dm^−3^)	21	38	596	118
K (mg dm^−3^)	28	185	3013	610
Ca (mg dm^−3^)	1574	319	1730	1314
Mg (mg dm^−3^)	131	75	625	167

## Data Availability

The data presented in this study are available on request from the corresponding author.

## Supplementary Materials

The following supporting information can be downloaded at: https://www.mdpi.com/article/10.3390/molecules30234545/s1, Table S1: The chemical composition of essential oils (%) obtained from inflorescences of English lavender ‘Sentivia Blue’ grown in peat amended with chemical fertilizer (P + F), peat amended with bark and sewage sludge compost (PBC), and peat amended with bark, sewage sludge compost, and chemical fertilizer (PBC + F); Table S2: The chemical composition of essential oils (%) obtained from leafy stems of English lavender ‘Sentivia Blue’ grown in peat amended with chemical fertilizer (P + F), peat amended with bark and sewage sludge compost (PBC), and peat amended with bark, sewage sludge compost, and chemical fertilizer (PBC + F); Table S3: Main effect of plant parts of English lavender ‘Sentivia Blue’ on the essential oils composition (%); Table S4: Main effect of growing media on the essential oils composition (%) of English lavender ‘Sentivia Blue’; Table S5: Physico-chemical and biological parameters of sewage sludge compost; Table S6: Chemical characteristics of growing media—a pilot study; Table S7: Plant growth parameters of English lavender ‘Sentivia Blue’ exposed to different media—a pilot study.
