# Supplementary material for: Changes in Growth and Chemical Composition of the Essential Oil from Flowers and Leafy Stems of Lavandula angustifolia Grown in Media Amended with Bark and Sewage Sludge
Source: Molecules. 2025 Nov 25;30(23):4545. doi: 10.3390/molecules30234545 (PMC12693513; doi:10.3390/molecules30234545)
Supplement: Supplementary file 1 [file molecules-30-04545-s001.zip › molecules-3972791-supplementary.pdf]

Supplementary Materials

Article

Changes in Growth and Chemical Composition of the Essential Oil from Flowers and Leafy Stems of *Lavandula angustifolia* Grown in Media Amended with Bark and Sewage Sludge

Agnieszka Zawadzińska [1,*], Aneta Wesołowska [2], Ewa Skutnik [3], Julita Rabiza-Świder [3] and Piotr Salachna [1]

[1] Department of Horticulture, West Pomeranian University of Technology in Szczecin, Słowackiego 17 Str., 71-434 Szczecin, Poland; piotr.salachna@zut.edu.pl (P.S.)
[2] Department of Organic and Physical Chemistry, Faculty of Chemical Technology and Engineering, West Pomeranian University of Technology in Szczecin, Piastów Ave. 42, 71-065 Szczecin, Poland; aneta.wesolowska@zut.edu.pl (A.W.)
[3] Section of Ornamental Plants, Institute of Horticultural Sciences, Warsaw University of Life Sciences, Nowoursynowska 166, 02-787 Warsaw, Poland; ewa_skutnik@sggw.edu.pl (E.S.); julita_rabiza_swider@sggw.edu.pl (J.R.-Ś.)
Correspondence: agnieszka.zawadzinska@zut.edu.pl; Tel.: +48-91-449-6391

Table S1. The chemical composition of essential oils (%) obtained from inflorescences of English lavender 'Sentivia Blue' grown in peat amended with chemical fertilizer (P + F), peat amended with bark and sewage sludge compost (PBC), and peat amended with bark, sewage sludge compost, and chemical fertilizer (PBC + F).

Table S2. The chemical composition of essential oils (%) obtained from leafy stems of English lavender 'Sentivia Blue' grown in peat amended with chemical fertilizer (P + F), peat amended with bark and sewage sludge compost (PBC), and peat amended with bark, sewage sludge compost, and chemical fertilizer (PBC + F).

Table S3. Main effect of plant parts of English lavender 'Sentivia Blue' on the essential oils composition (%).

Table S4. Main effect of growing media on the essential oils composition (%) of English lavender 'Sentivia Blue'.

Table S5. Physico-chemical and biological parameters of sewage sludge compost.

Table S6. Chemical characteristics of growing media - a pilot study.

Table S7. Plant growth parameters of English lavender 'Sentivia Blue' exposed to different media - a pilot study.

Table S1. The chemical composition of essential oils (%) obtained from inflorescences of English lavender 'Sentivia Blue' grown in peat amended with chemical fertilizer (P + F), peat amended with bark and sewage sludge compost (PBC), and peat amended with bark, sewage sludge compost, and chemical fertilizer (PBC + F). Rt—retention time; RI—retention indices relative to n-alkanes (C7–C30) on HP-5 MS capillary column.

No.	Compound	Rt[min]	RI	Growing Media		
				P + F	PBC	PBC + F
1.	α-Pinene	6.21	931	0.55	0.40	0.41
2.	Camphene	6.57	946	0.14	0.11	0.12
3.	Thuja-2,4(10)-diene	6.70	952	0.05	0.05	0.05
4.	β-Thujene	7.13	969	0.12	0.11	0.10
5.	Sabinene	7.20	971	0.05	0.03	0.03
6.	β-Pinene	7.27	974	1.40	1.21	1.16
7.	3-Octanone	7.52	984	0.81	0.36	0.52
8.	β-Myrcene	7.68	991	0.54	0.60	0.58
9.	3-Octanol	7.76	994	0.19	0.10	0.12
10.	α-Phellandrene	8.08	1006	0.29	0.28	0.27
11.	δ-3-Carene	8.15	1008	0.08	0.07	0.06
12.	α-Terpinene	8.30	1014	0.41	0.32	0.34
13.	m-Cymene	8.47	1020	0.41	0.44	0.39
14.	p-Cymene	8.54	1022	0.79	0.85	0.74
15.	D-Limonene	8.65	1026	0.23	0.23	0.20
16.	Eucalyptol	8.71	1028	1.11	1.23	1.39
17.	(Z)-β-Ocimene	8.91	1035	0.11	0.17	0.15
18.	(E)-β-Ocimene	9.19	1045	0.16	0.26	0.24
19.	γ-Terpinene	9.48	1055	0.09	0.10	0.09
20.	cis-β-Terpineol	9.71	1064	0.09	0.06	0.08
21.	cis-Linalool oxide	9.89	1070	5.52	4.49	5.17
22.	trans-Linalool oxide	10.17	1080	0.12	0.13	0.12
23.	α-Terpinolene	10.32	1085	4.25	3.39	3.93
24.	Linalool	10.77	1101	15.17	17.13	17.60
25.	Hotrienol	10.83	1103	2.00	1.32	1.61
26.	1-Octen-3-yl acetate	10.97	1108	0.59	0.29	0.37
27.	α-Thujone	11.07	1112	0.15	0.17	0.20
28.	Fenchol	11.12	1114	0.16	0.18	0.15
29.	Phenethyl alcohol	11.22	1118	0.06	0.10	0.11
30.	Myrcenol	11.25	1119	0.17	0.15	0.13
31.	3-Octanol acetate	11.37	1123	0.20	0.22	0.21
32.	α-Campholenal	11.49	1128	0.24	0.20	0.22
33.	4-Acetyl-1-methylcyclohexene	11.62	1132	0.07	0.04	0.07
34.	cis-p-Mentha-2,8-dien-1-ol	11.68	1135	-	-	0.18
35.	1,2-Dihydrolinalool	11.74	1137	1.65	2.00	1.67
36.	trans-Pinocarveol	11.81	1139	0.21	0.19	0.20
37.	Camphor	11.89	1142	0.84	0.81	0.82
38.	Lilac aldehyde C	12.06	1149	0.25	0.19	0.23
39.	Nerol oxide	12.16	1152	0.64	0.56	0.57
40.	(E,Z)-2,6-Nonadienal	12.24	1155	0.18	0.18	0.17
41.	Pinocarvone	12.39	1161	0.96	1.00	0.86
42.	Borneol	12.50	1165	3.73	3.26	3.41
43.	p-Mentha-1,5-dien-8-ol	12.71	1172	0.61	0.43	0.52

44.	Terpinen-4-ol	12.81	1176	0.33	0.38	0.34
45.	p-Cymen-8-ol	12.94	1181	0.71	0.68	0.67
46.	Thuj-3-en-1-ol	13.04	1185	0.63	0.57	0.59
47.	α-Terpineol	13.19	1190	1.05	1.51	1.50
48.	Myrtenol	13.34	1196	1.68	1.82	1.62
49.	Dodecane	13.46	1200	0.30	0.30	0.28
50.	Verbenone	13.67	1208	1.46	1.42	1.37
51.	(E,E)-2,4-Nonadienal	13.90	1216	0.20	0.18	0.19
52.	cis-Carveol	13.96	1219	0.15	0.19	0.17
53.	trans-Carveol	14.05	1222	0.26	0.27	0.28
54.	Bornyl formate	14.20	1228	0.26	0.36	0.31
55.	Citronellol	14.26	1230	0.39	0.38	0.41
56.	p-Cumic aldehyde	14.50	1239	0.33	0.34	0.33
57.	D-Carvone	14.65	1244	0.14	0.19	0.16
58.	trans-Geraniol	14.86	1252	0.09	0.12	-
59.	Linalyl acetate	14.99	1257	9.59	8.01	8.79
60.	α-Citral	15.33	1270	0.13	0.18	0.18
61.	Phellandral	15.57	1278	0.16	0.17	0.19
62.	trans-Bornyl acetate	15.79	1287	0.12	0.14	0.13
63.	Lavandulyl acetate	15.90	1291	3.07	3.39	3.20
64.	Tridecane	16.15	1300	0.37	0.42	0.41
65.	Undecanal	16.38	1309	0.06	0.14	0.14
66.	neo-Verbanol acetate	16.83	1326	-	0.08	0.08
67.	Piperitenone	17.18	1339	0.38	0.35	0.37
68.	α-Cubebene	17.45	1350	-	0.06	-
69.	α-Terpineol acetate	17.56	1354	0.22	0.12	0.18
70.	Nerol acetate	17.84	1365	0.35	0.53	0.48
71.	α-Copaene	18.16	1377	0.25	0.14	0.16
72.	Geranyl acetate	18.35	1384	1.44	1.77	1.47
73.	1-Tetradecene	18.71	1398	0.32	0.12	0.20
74.	cis-α-Bergamotene	19.16	1416	0.16	0.10	0.08
75.	α-Santalene	19.28	1421	1.33	1.16	0.94
76.	trans-α-Bergamotene	19.67	1437	0.17	0.12	0.10
77.	Aromadendrene	19.91	1446	0.08	0.06	0.05
78.	Epi-β-Santalene	19.97	1448	0.08	0.07	0.06
79.	α-Humulene	20.26	1460	0.08	0.08	0.07
80.	Di-epi-α-Cedrene	20.45	1468	0.05	-	-
81.	1-Dodecanol	20.66	1476	0.06	-	-
82.	γ-Muurolene	20.82	1483	0.10	0.06	0.06
83.	Germacrene D	20.88	1485	0.07	0.06	0.05
84.	α-Bulnesene	21.40	1506	-	0.04	0.04
85.	γ-Cadinene	21.61	1515	0.69	0.48	0.37
86.	β-Sesquiphellandrene	21.71	1520	0.74	0.81	0.73
87.	trans-Calamenene	22.04	1534	0.12	0.07	0.08
88.	α-Cadinene	22.29	1544	0.19	0.14	0.12
89.	α-Calacorene	22.36	1547	-	0.06	0.05
90.	Elemol	22.52	1554	1.11	1.32	1.17
91.	β-Calacorene	22.68	1561	0.04	0.07	0.07
92.	E-Nerolidol	22.78	1565	0.22	0.16	0.17
93.	(Z)-3-Hexenyl benzoate	23.07	1577	0.14	0.13	0.13

94.	Caryophyllene oxide	23.32	1588	11.09	12.72	11.92
95.	Viridiflorol	23.43	1593	0.17	0.18	0.19
96.	1-Hexadecene	23.50	1596	0.13	0.15	0.13
97.	Hexadecane	23.66	1603	0.26	0.30	0.31
98.	Cedrol	23.85	1611	0.53	0.61	0.57
99.	Longifolenaldehyde	23.96	1616	0.50	0.47	0.47
100.	Di-epi-1,10-cubenol	24.13	1624	0.49	0.35	0.43
101.	Isospathulenol	24.37	1634	0.33	0.36	0.37
102.	Caryophylla-4(12),8(13)-dien-5β-ol	24.46	1638	0.77	1.06	0.97
103.	τ-Cadinol	24.55	1642	2.11	1.78	1.54
104.	α-Muurolol	24.85	1656	-	-	0.33
105.	α-Eudesmol	24.93	1659	2.13	2.39	2.10
106.	9-Cedranone	25.10	1667	0.22	0.18	0.20
107.	Cadalene	25.24	1673	2.08	2.80	2.50
108.	(Z)-α-Bisabolene epoxide	25.39	1680	0.35	0.43	0.37
109.	α-Bisabolol	25.47	1683	0.62	0.44	0.57
110.	epi-α-Bisabolol	25.60	1689	0.61	0.45	0.57
111.	Heptadecane	25.96	1706	0.12	0.10	0.12
112.	5-Ethyl-5-methylpentadecane	26.08	1711	0.25	0.30	0.30
113.	Pentadecanal	26.19	1716	0.33	0.27	0.26
114.	(2E,6E)-Farnesol	26.82	1746	0.40	0.39	0.36
115.	Santalol	27.29	1768	0.24	0.25	0.22
116.	(E)-α-Atlantone	27.42	1774	-	0.09	0.07
117.	1-Octadecene	27.94	1799	0.07	0.11	0.06
118.	Octadecane	27.99	1801	0.09	-	-
119.	Hexahydrofarnesyl acetone	28.87	1844	-	0.19	0.13
120.	2-Methylanthracene	30.61	1930	-	0.07	0.04
121.	Palmitic acid	31.34	1967	-	0.08	-
122.	1-Heneicosene	34.31	2091	-	0.14	-
	**Total identified**			**99.15**	**99.39**	**99.52**

Table S2. The chemical composition of essential oils (%) obtained from leafy stems of English lavender 'Sentivia Blue' grown in peat amended with chemical fertilizer (P + F), peat amended with bark and sewage sludge compost (PBC), and peat amended with bark, sewage sludge compost, and chemical fertilizer (PBC + F). Rt—retention time; RI—retention indices relative to n-alkanes (C7–C30) on HP-5 MS capillary column.

No.	Compound	Rt[min]	RI	Growing Media		
				P + F	PBC	PBC + F
1.	α-Thujene	6.04	924	0.07	-	0.05
2.	α-Pinene	6.20	931	0.94	0.78	0.75
3	Camphene	6.56	946	0.38	0.28	0.32
4.	Thuja-2,4(10)-diene	6.70	952	0.05	-	0.06
5.	β-Thujene	7.12	968	0.57	0.65	0.56
6.	Sabinene	7.19	971	0.20	-	0.07
7.	β-Pinene	7.28	975	3.74	3.09	2.98
8.	3-Octanone	7.52	984	0.17	0.09	0.15
9.	β-Myrcene	7.67	990	0.26	0.23	0.29
10.	α-Phellandrene	8.08	1006	-	0.08	0.08
11.	δ-3-Carene	8.15	1008	0.84	0.71	0.66
12.	α-Terpinene	8.32	1014	0.15	0.14	0.17
13.	m-Cymene	8.47	1020	1.12	1.12	1.23
14.	p-Cymene	8.54	1022	2.23	2.34	2.37
15.	D-Limonene	8.65	1026	0.90	0.75	0.81
16.	Eucalyptol	8.71	1028	1.53	1.32	1.40
17.	(Z)-β-Ocimene	8.90	1035	-	-	0.05
18.	(E)-β-Ocimene	9.19	1045	0.08	-	0.13
19.	γ-Terpinene	9.47	1055	0.26	0.23	0.27
20.	*cis*-β-Terpineol	9.71	1064	0.12	0.10	0.08
21.	*cis*-Linalool oxide	9.85	1069	0.48	0.53	0.63
22.	*trans*-Linalool oxide	10.15	1079	0.20	0.21	0.22
23.	α-Terpinolene	10.30	1084	0.62	0.69	0.74
24.	2-Nonanone	10.51	1092	0.14	0.12	0.16
25.	Linalool	10.77	1101	3.77	3.64	4.15
26.	1-Octen-3-yl acetate	10.96	1108	0.31	0.26	0.26
27.	α-Thujone	11.09	1113	0.11	0.13	0.15
28.	Phenethyl alcohol	11.22	1118	0.20	0.22	0.17
29.	3-Octanol acetate	11.36	1123	0.38	0.38	0.37
30.	4-Acetyl-1-methylcyclohexene	11.62	1132	0.25	-	-
31.	1,2-Dihydrolinalool	11.73	1137	2.34	2.61	2.23
32.	Camphor	11.87	1142	1.89	1.52	1.57
33.	Lilac aldehyde A	11.99	1146	-	0.12	0.15
34.	(E,Z)-2,6-Nonadienal	12.21	1154	0.20	0.19	0.18
35.	Pinocarvone	12.39	1161	2.35	2.29	2.12
36.	Borneol	12.55	1167	9.38	7.13	6.92
37.	Terpinen-4-ol	12.80	1176	0.88	0.88	0.69
38.	p-Cymen-8-ol	12.95	1182	1.04	1.33	1.30
39.	Thuj-3-en-1-ol	13.04	1185	0.77	0.96	0.87
40.	α-Terpineol	13.18	1190	0.60	0.65	0.63
41.	Myrtenol	13.34	1196	2.93	2.91	2.59
42.	Verbenone	13.67	1208	1.26	1.55	1.40
43.	(E,E)-2,4-Nonadienal	13.87	1215	-	0.09	0.12

44.	*cis*-Carveol	13.96	1219	0.38	0.53	0.31
45.	*trans*-Carveol	14.09	1223	0.28	0.33	0.24
46.	Bornyl formate	14.20	1228	1.13	1.04	1.06
47.	p-Cumic aldehyde	14.54	1240	0.19	0.26	0.29
48.	D-Carvone	14.65	1244	0.38	0.41	0.35
49.	*trans*-Geraniol	14.86	1252	0.32	0.37	0.32
50.	Linalyl acetate	14.94	1255	1.47	1.77	1.93
51.	α-Citral	15.33	1270	0.23	0.30	0.25
52.	Phellandral	15.50	1276	0.13	0.13	0.10
53.	*trans*-Bornyl acetate	15.77	1286	0.39	0.31	0.39
54.	Lavandulyl acetate	15.89	1290	0.77	1.02	1.09
55.	Thymol	16.00	1294	0.15	0.16	0.19
56.	Menthyl acetate	16.08	1297	-	-	0.21
57.	Tridecane	16.14	1300	0.44	0.55	0.24
58.	*cis*-Pinocarvyl acetate	16.25	1304	0.19	0.25	0.40
59.	Carvacrol	16.49	1313	0.20	0.20	0.20
60.	neo-Verbanol acetate	16.82	1326	0.09	0.11	0.12
61.	δ-Elemene	16.93	1330	0.12	0.09	-
62.	Piperitenone	17.18	1339	0.51	0.62	0.63
63.	α-Terpineol acetate	17.55	1354	0.07	0.08	0.09
64.	Nerol acetate	17.82	1364	0.42	0.27	0.31
65.	α-Copaene	18.16	1377	0.12	0.09	0.09
66.	Geranyl acetate	18.35	1384	5.93	5.62	5.69
67.	β-Elemene	18.58	1393	0.31	-	0.13
68.	1-Tetradecene	18.71	1398	-	0.09	-
69.	*cis*-α-Bergamotene	19.15	1416	0.31	0.31	0.28
70.	α-Santalene	19.28	1421	2.19	2.53	2.14
71.	*trans*-α-Bergamotene	19.65	1436	0.41	0.43	0.37
72.	α-Guaiene	19.74	1439	0.10	-	-
73.	Aromadendrene	19.90	1446	0.20	0.22	0.19
74.	Epi-β-Santalene	19.96	1448	0.15	0.17	0.15
75.	(E)-Geranyl acetone	20.16	1456	0.10	-	0.09
76.	α-Humulene	20.22	1459	0.14	0.25	0.16
77.	γ-Muurolene	20.81	1482	0.13	-	-
78.	Germacrene D	20.89	1486	0.28	0.33	0.35
79.	Pentadecane	21.25	1500	0.08	0.06	0.06
80.	α-Bulnesene	21.40	1506	0.27	-	0.13
81.	β-Bisabolene	21.45	1509	-	0.06	-
82.	γ-Cadinene	21.61	1515	1.82	1.61	1.61
83.	β-Sesquiphellandrene	21.72	1520	1.36	1.62	1.80
84.	*cis*-Calamenene	21.81	1524	0.17	0.13	-
85.	*trans*-Cadina-1,4-diene	21.96	1530	0.51	0.48	0.45
86.	*trans*-Calamenene	22.05	1534	0.56	0.49	0.48
87.	α-Cadinene	22.30	1545	0.54	0.54	0.51
88.	Elemol	22.52	1554	1.14	1.30	1.25
89.	β-Calacorene	22.66	1560	0.10	0.10	0.12
90.	E-Nerolidol	22.76	1565	-	-	0.12
91.	Dodecanoic acid	22.81	1566	0.43	0.40	0.33
92.	(Z)-3-Hexenyl benzoate	23.06	1577	0.29	0.24	0.29
93.	Caryophyllene oxide	23.31	1588	10.20	11.77	10.86

94.	Viridiflorol	23.43	1593	0.14	0.17	0.28
95.	1-Hexadecene	23.54	1597	0.29	0.27	0.46
96.	Hexadecane	23.66	1603	0.25	0.28	0.43
97.	Cedrol	23.85	1611	0.70	0.73	0.67
98.	Longifolenaldehyde	23.97	1616	0.83	0.77	0.73
99.	epi-Cubenol	24.08	1621	0.20	0.21	0.23
100.	Di-epi-1,10-cubenol	24.15	1625	0.21	0.29	0.41
101.	γ-Eudesmol	24.20	1627	-	0.12	-
102.	Isospathulenol	24.37	1634	0.26	0.34	-
103.	Caryophylla-4(12),8(13)-dien-5β-ol	24.46	1638	0.48	0.71	0.19
104.	τ-Cadinol	24.58	1644	5.64	4.91	5.61
105.	Cubenol	24.76	1652	0.57	0.61	0.56
106.	α-Muurolol	24.86	1656	0.39	0.38	0.56
107.	α-Eudesmol	24.92	1659	0.90	1.31	1.28
108.	α-Cadinol	25.02	1663	0.60	0.50	-
109.	Cadalene	25.23	1673	2.25	2.67	2.21
110.	1-Tetradecanol	25.33	1678	0.30	0.31	0.13
111.	(Z)-α-Bisabolene epoxide	25.39	1680	0.34	0.44	0.82
112.	α-Bisabolol	25.47	1683	-	0.22	0.47
113.	epi-α-Bisabolol	25.60	1689	1.52	1.40	1.53
114.	Heptadecane	25.94	1705	0.22	0.25	0.32
115.	5-Ethyl-5-methylpentadecane	26.06	1710	0.48	0.54	0.20
116.	Pentadecanal	26.18	1716	0.61	0.57	0.42
117.	(2Z,6E)-Farnesol	26.30	1722	-	-	0.20
118.	Methyl tetradecanoate	26.47	1730	0.21	0.19	-
119.	(2E,6E)-Farnesol	26.80	1745	0.89	0.80	0.98
120.	Santalol	27.29	1768	0.22	0.30	0.44
121.	(E)-α-Atlantone	27.40	1773	0.12	0.12	0.13
122.	Phenantrene	27.64	1785	0.11	0.11	0.17
123.	1-Octadecene	27.90	1798	-	-	0.13
124.	Octadecane	27.97	1800	0.19	0.18	0.10
125.	Hexadecanal	28.12	1807	0.12	0.14	-
126.	Hexahydrofarnesyl acetone	28.84	1843	0.11	0.16	0.27
127.	Pentadecanoic acid	29.35	1868	0.08	0.12	0.22
128.	Isophytol	31.05	1952	0.07	0.08	0.08
129.	m-Camphorene	31.17	1958	0.84	0.77	1.55
130.	1-Heptadecanol	31.59	1979	0.11	0.13	0.15
131.	1-Eicosene	31.97	1998	0.09	0.07	-
132.	5-Ethylnonadecane	32.81	2032	0.08	0.09	0.11
133.	1-Octadecanol	34.05	2081	0.11	0.14	0.15
	**Total identified**			**99.94**	**99.36**	**98.66**

Table S3. Main effect of plant parts of English lavender 'Sentivia Blue' on the essential oils composition (%). The data are presented as mean ± SD.

Compound	Plant Part	
	Inflorescences	Leafy Stems
β-Pinene ***	1.25 ± 0.13	3.27 ± 0.69
m-Cymene ***	0.41 ± 0.04	1.15 ± 0.14
p-Cymene ***	0.79 ± 0.07	2.31 ± 0.30
Eucalyptol ns	1.24 ± 0.14	1.41 ± 0.21
cis-Linalool oxide ***	5.06 ± 0.49	0.55 ± 0.08
α-Terpinolene ***	3.88 ± 0.45	0.14 ± 0.02
Linalool ***	16.63 ± 1.24	3.85 ± 0.37
1,2-Dihydrolinalool **	1.77 ± 0.21	2.39 ± 0.22
Camphor ***	0.82 ± 0.03	1.66 ± 0.24
Pinocarvone***	0.94 ± 0.07	2.25 ± 0.25
Borneol ***	3.46 ± 0.22	7.81 ± 1.36
p-Cymen-8-ol ***	0.69 ± 0.06	1.22 ± 0.19
α-Terpineol ***	1.35 ± 0.24	0.62 ± 0.08
Myrtenol ***	1.70 ± 0.10	2.81 ± 0.28
Verbenone ns	1.42 ± 0.07	1.40 ± 0.19
Bornyl formate ***	0.31 ± 0.06	1.07 ± 0.13
Linalyl acetate ***	8.80 ± 0.82	1.72 ± 0.30
Lavandulyl acetate ***	3.22 ± 0.16	0.96 ± 0.16
Geranyl acetate ***	1.56 ± 0.21	5.74 ± 0.27
α-Santalene ***	1.14 ± 0.18	2.29 ± 0.20
γ-Cadinene ***	0.51 ± 0.15	1.68 ± 0.12
β-Sesquiphellandrene ***	0.76 ± 0.07	1.59 ± 0.20
Elemol ns	1.20 ± 0.12	1.23 ± 0.11
Caryophyllene oxide *	11.91 ± 0.89	10.94 ± 0.80
τ-Cadinol ***	1.81 ± 0.28	5.38 ± 0.74
α-Eudesmol ***	2.21 ± 0.17	1.16 ± 0.23
Cadalene ns	2.46 ± 0.34	2.37 ± 0.31
epi-α-Bisabolol **	0.54 ± 0.08	1.48 ± 0.42

NS,***, **, *, Nonsignificant or significant F test at $p \leq 0.001$, 0.01 or 0.05, respectively.

Table S4. Main effect of growing media on the essential oils composition (%) of English lavender 'Sentivia Blue'. The data are presented as mean ± SD. Peat + chemical fertilizer (P + F); 40% peat + 50% bark + 10% sewage sludge compost (PBC); 40% peat + 50% bark + 10% sewage sludge compost + chemical fertilizer (PBC + F).

Compound	Growing Media		
	**P + F**	**PBC**	**PBC + F**
β-Pinene ns	2.57 ± 1.35	2.15 ± 1.29	2.07 ± 1.09
m-Cymene ns	0.76 ± 0.41	0.78 ± 0.42	0.81 ± 0.49
p-Cymene ns	1.51 ± 0.83	1.59 ± 0.92	1.55 ± 0.96
Eucalyptol ns	1.32 ± 0.26	1.28 ± 0.21	1.39 ± 0.12
cis-Linalool oxide **	3.00 ± 2.91 a	2.51 ± 2.29 b	2.90 ± 2.62 a
α-Terpinolene **	2.24 ± 2.43 a	1.76 ± 1.89 b	2.05 ± 2.18 ab
Linalool *	9.47 ± 6.58 b	10.39 ± 7.81 ab	10.87 ± 7.78 a
1,2-Dihydrolinalool *	1.99 ± 0.43 ab	2.30 ± 0.36 a	1.95 ± 0.36 b
Camphor ns	1.36 ± 0.61	1.16 ± 0.42	1.19 ± 0.47
Pinocarvone ns	1.65 ± 0.81	1.64 ± 0.76	1.49 ± 0.76
Borneol *	6.56 ± 3.29 a	5.19 ± 2.25 b	5.16 ± 2.11 b
p-Cymen-8-ol ns	0.87 ± 0.20	1.01 ± 0.38	0.98 ± 0.40
α-Terpineol **	0.82 ± 0.26 b	1.08 ± 0.49 a	1.06 ± 0.51 a
Myrtenol ns	2.30 ± 0.73	2.11 ± 0.60	1.68 ± 0.04
Verbenone ns	1.36 ± 0.14	1.48 ± 0.09	1.38 ± 0.16
Bornyl formate ns	0.69 ± 0.50	0.70 ± 0.39	0.69 ± 0.46
Linalyl acetate ns	5.53 ± 4.70	4.89 ± 3.60	5.38 ± 3.99
Lavandulyl acetate **	1.91 ± 1.33 b	2.21 ± 1.40 a	2.14 ± 1.23 a
Geranyl acetate ns	3.68 ± 2.59	3.69 ± 2.23	3.58 ± 2.45
α-Santalene **	1.76 ± 0.50 a	1.84 ± 0.80 a	1.54 ± 0.69 b
γ-Cadinene ***	1.26 ± 0.65 a	1.04 ± 0.65 b	0.99 ± 0.72 b
β-Sesquiphellandrene **	1.05 ± 0.36 b	1.21 ± 0.48 a	1.26 ± 0.62 a
Elemol ns	1.12 ± 0.06	1.31 ± 0.11	1.21 ± 0.06
Caryophyllene oxide *	10.64 ± 0.68 b	12.24 ± 0.72 a	11.39 ± 0.75 ab
τ-Cadinol ns	3.87 ± 2.06	3.34 ± 1.81	3.57 ± 2.48
α-Eudesmol *	1.52 ± 0.71 b	1.85 ± 0.63 a	1.69 ± 0.49 ab
Cadalene *	2.16 ± 0.20 b	2.73 ± 0.16 a	2.35 ± 0.26 ab
epi-α-Bisabolol ns	1.06 ± 0.53	0.92 ± 0.56	1.05 ± 0.75

NS,***, **, *, Nonsignificant or significant F test at $p \leq 0.001$, 0.01 or 0.05, respectively. Values in raw with different letters show statistically significant differences based on ANOVA and Tukey test at $p \leq 0.05$.

Table S5. Physico-chemical and biological parameters of sewage sludge compost.

Parameters	Compost	References Value*
Bulk density (g cm^{-1})	0.65	-
Humidity (%)	40.4	-
Organic matter (%)	43.1	≥ 30
Total N (%) DW	1.69	≥ 0.3
P_2O_5 (%) DW	0.32	≥ 0.2
K_2O (%) DW	0.29	≥ 0.2
CaO (%) DW	3.09	-
Mg (%) DW	0.37	-
Zn (mg kg^{-1}) DW	277	-
Cu (mg kg^{-1}) DW	37.2	-
Cd (mg kg^{-1}) DW	0.98	5.0
Pb (mg kg^{-1}) DW	29.4	140
Ni (mg kg^{-1}) DW	18.2	60
Cr (mg kg^{-1}) DW	43.2	100
Hg (mg kg^{-1}) DW	< 0.05	2.0
Salmonella sp.	absent	absent
Ascaris sp., *Trichuris* sp., *Toxocara* sp.	absent	absent

*according to Regulation of the Minister of Agriculture and Rural Development of 9 August 2024 on the implementation of certain provisions of the Act on Fertilizers and Fertilization, *Journal of Laws* **2024**, *item* 1261. (In Polish)

Table S6. Chemical characteristics of growing media - a pilot study.

Media	N-NO_3 (mg dm^{-3})	P (mg dm^{-3})	K (mg dm^{-3})	Salinity (g NaCl dm^{-3})
PBC_{10}*	21	119	574	0.72
PBC_{20}	30	165	886	1.45
PBC_{30}	46	211	1065	1.74

*PBC_{10}, 40% peat + 50% bark + 10% sewage sludge compost; PBC_{20}, 30% peat + 50% bark + 20% sewage sludge compost; PBC_{30}, 20% peat + 50% bark + 30% sewage sludge compost.

Table S7. Plant growth parameters of English lavender 'Sentivia Blue' exposed to different media - a pilot study.

Media	Plant Height (cm)	Plant Width (cm)	Leafy Stem Number per Plant	Leaf Greenness Index SPAD	Plant Fresh Weight (g)
PBC_{10}*	24.7	26.5	19.5	54.5	29.1
PBC_{20}	24.3	12.5	8.30	32.7	9.58
PBC_{30}	18.5	16.7	5.33	29.2	7.70

*PBC_{10}, 40% peat + 50% bark + 10% sewage sludge compost; PBC_{20}, 30% peat + 50% bark + 20% sewage sludge compost; PBC_{30}, 20% peat + 50% bark + 30% sewage sludge compost.
